# Comparative Neuroanatomical Study of the Main Olfactory Bulb in Domestic and Wild Canids: Dog, Wolf and Red Fox

**DOI:** 10.3390/ani12091079

**Published:** 2022-04-21

**Authors:** Irene Ortiz-Leal, Mateo V. Torres, Linda Noa López-Callejo, Luis Eusebio Fidalgo, Ana López-Beceiro, Pablo Sanchez-Quinteiro

**Affiliations:** Department of Anatomy, Animal Production and Clinical Veterinary Sciences, Faculty of Veterinary, University of Santiago de Compostela, Av. Carballo Calero s/n, 27002 Lugo, Spain; irene.ortiz.leal@usc.es (I.O.-L.); mateovazquez.torres@usc.es (M.V.T.); noa1196@hotmail.com (L.N.L.-C.); luis.fidalgo@usc.es (L.E.F.); anam.lopez.beceiro@usc.es (A.L.-B.)

**Keywords:** Canidae, olfactory system, immunohistochemistry, lectins, domestication

## Abstract

**Simple Summary:**

The study of the morphological, physiological and molecular changes associated with the domestication process has been one of the most interesting unresolved neuroanatomical issues. The olfactory system deserves special attention since both wild and domestic canids are macrosmatic mammals with very high olfactory capacities. Nevertheless, the question remains open as to whether domestication involuted the sense of smell in domestic dogs. Further, there is a lack of comparative morphological information on the olfactory bulb, the first structure integrating olfactory sensory information in the brain. To provide comparative information on the domestication process, we studied the olfactory bulb of dogs and their two most important wild ancestors: the wolf and the fox. The study was carried out by macroscopic dissection and histological and immunohistochemical techniques and has allowed us to verify, first of all, that the three species present olfactory bulbs corresponding to a macrosmatic animal, but that there are noticeable differences not only in size, which was already known, but also in the cellularity and intensity of the immunohistochemical pattern characteristic of each species. These variations point to a reduction of the olfactory system as a consequence of the selection pressure associated with the domestication of animals.

**Abstract:**

The sense of smell plays a fundamental role in mammalian survival. There is a considerable amount of information available on the vomeronasal system of both domestic and wild canids. However, much less information is available on the canid main olfactory system, particularly at the level of the main olfactory bulb. Comparative study of the neuroanatomy of wild and domestic canids provides an excellent model for understanding the effects of selection pressure associated with domestication. A comprehensive histological (hematoxylin–eosin, Nissl, Tolivia and Gallego’s Trichrome stains), lectin (UEA, LEA) and immunohistochemical (Gαo, Gαi2, calretinin, calbindin, olfactory marker protein, glial fibrillary acidic protein, microtubule-associated protein 2) study of the olfactory bulbs of the dog, fox and wolf was performed. Our study found greater macroscopic development of the olfactory bulb in both the wolf and fox compared to the dog. At the microscopic level, all three species show a well-developed pattern of lamination and cellularity typical of a macrosmatic animal. However, greater development of cellularity in the periglomerular and mitral layers of wild canids is characteristic. Likewise, the immunohistochemical study shows comparable results between the three species, but with a noticeably higher expression of markers in wild canids. These results suggest that the reduction in encephalization experienced in dogs due to domestication also corresponds to a lower degree of morphological and neurochemical differentiation of the olfactory bulb.

## 1. Introduction

Among all mammalian sensory systems, the sense of smell plays a fundamental role in mammalian survival, especially in terms of reproduction, food selection, recognition of predators and conspecifics [1,2,3]. In the case of Canidae, the olfactory capability is extraordinary, as much as 10,000–100,000 times that of the average human, and has a lower limit of detectability for volatile compounds of one part per trillion [4,5]. Olfaction involves two primary systems: the main olfactory system (MOS) and the accessory olfactory system, also known as the vomeronasal system (VNS). The olfactory epithelium (OE) of the MOS senses a large array of odorants, mainly volatile, which generate a conscious response, the smell [6,7,8]. These compounds are detected by the neuroreceptor cells of the main olfactory epithelium (MOE) which project to the main integrative center of the OS, the main olfactory bulb (MOB) [9,10]. In contrast, the VNS responds to critical signals for sexual and social behaviors [11,12], but without producing a conscious perception of the chemical molecules detected, mainly pheromones and kairomones [13,14,15]. The VNS system comprises a tubular sensory structure, the vomeronasal organ (VNO) [16,17], and its first integrative center in the brain is the accessory olfactory bulb (AOB) [18,19].

There is a considerable amount of neuroanatomical and neurochemical information available on the receptor and integrative structures of the vomeronasal system at the level of the VNO [20,21] and the AOB [22,23,24] in both domestic and wild canids. However, much less information is available on the canid MOS, particularly at the level of the first integrative center, the MOB [25]. To our knowledge, only classical neuroanatomical studies such as those carried out by Jawlowski [26] and Miodonski [27] in the dog and fox and more recent studies on the immunohistochemistry of calcium-binding proteins in the dog MOB [28] and the morphological and neurochemical characterization of the olfactory bulb of the African wild dog [29] are noteworthy.

The comparative study of the neuroanatomy of wild and domestic canids has the additional interest of providing an excellent model for assessing and understanding the effects of selection pressure associated with domestication on the configuration of neural structures that support species-specific behaviors [30,31]. In the case of the olfactory system, the study by Bird et al. [32] suggests that there is a loss of olfactory capacity in dogs compared to two wild canids, the coyote and gray wolf. This conclusion is based on morphometric analysis of the cribriform plate (CP) surface area relative to body size, as these authors previously demonstrated that the relative CP size is closely correlated with the number of olfactory receptor genes in a species’ genome [33]. In the case of the structural configuration of the olfactory bulb, there is, to our knowledge, no comparative study of the effects of domestication. However, there is a significant precedent in a comparative study of the accessory olfactory bulb of the dog and fox [34], in which remarkable differences in cellularity, lamination and neurochemical properties have been found that point to involution as a consequence of domestication.

We have undertaken an exhaustive comparative study of the main olfactory bulb of the three most-relevant species of the Canidae family—dog, fox and wolf—in order to fill the existing gaps in the neuroanatomical study of the main olfactory bulb of canids and to evaluate the existence of qualitative differences that could support a hypothetical involution of the main olfactory bulb as a consequence of domestication.

## 2. Materials and Methods

### 2.1. Samples

Three red foxes (*Vulpes vulpes*), three wolves (*Canis lupus signatus*) and three dogs (*Canis lupus familiaris*) were employed for this study. All of them were adult males. The foxes came from activities organized by the Galician Hunting Federation. They were obtained in the field, the same day of their shooting, with a maximum of two hours delay. The wolves came from wildlife recovery centers in the Galician provinces where they had arrived after fatal traumatic accidents. We used individuals that had died recently, and only those without external or internal head injuries were selected. All samples of foxes and wolves were employed with the necessary permissions by the Galician Environment, Territory and Tenement Council (CMATV approval numbers EB-009/2020 and EB-007/2021). The three dogs necropsied were adult mesaticephalic dogs that came from the Department of Clinical Sciences of our School, where they had died due to clinical conditions. They were two German shepherds and one mastiff. The heads were intact and did not show clinical or postmortem evidence of neurological disease.

The whole brains containing the olfactory bulbs were extracted after opening dorsally the cranium and removing the lateral walls of the ethmoidal fossa with the help of an electric plaster cutter and a gouge clamp. In two animals of each species, the brains were preserved in Bouin’s fixative for 24 h, and afterwards, they were transferred to 70% ethanol. The remaining brain was fixed in 10% formalin. Prior to sectioning, the brains were photographed to show the lateral, dorsal and ventral aspects. The olfactory bulbs were dissected out. The left and right OB from every single animal was separately embedded in paraffin wax and serially cut, one sectioned transversely and the other sagittaly by a microtome with a thickness of 8 µm. The sections were mounted onto gelatin-coated slides, dewaxed and stained using haematoxylin-eosin, Nissl, Tolivia and Gallego’s trichrome stains, and lectin histochemical and immunohistochemical techniques. For each of the species studied, the three animals sampled showed an analogous pattern in both their macroscopic and microscopic morphology at the level of lamination, cellularity and their reaction to histological staining, as well as in the pattern of the immunohistochemical and histochemical lectin labeling. The slides were examined and evaluated blindly by two investigators.

### 2.2. Tolivia Protocol

The procedure followed is explained in detail in Tolivia et al. [35]. Briefly, after deparaffinizing and hydrating, sections were mordanted for 1 h in 2.5% FeNH_4_(SO_4_)_2_. After 2.5 h, the sections were rinsed in a freshly made solution: 50 mL of 50% ethanol to which 5 mL of 20% hematoxylin and 10 mL of 1% Li_2_CO_3_ were added. Finally, sections were washed for 3 × 5 min prior to staining for 5 min in the following solution: 0.2% pyronine and 20% formaldehyde.

### 2.3. Gallego’s Trichrome Protocol

This stain allows for the differentiation of components of the connective tissue. It stains erythrocytes green, muscle fibers and collagen light blue, epithelium and glandular tissue red, bone dark blue and cartilage purple. The protocol used was described in detail in Villamayor et al. [36] as follows: sections were first stained with Ziehl acetic fuchsin for 2 min. After several washes they were immersed into formalin–acetic acid solution for 5 min. After two more washes, the sections were finally stained with picroindigocarmine for 3–5 min.

### 2.4. Immunohistochemical Protocol

Antigen retrieval was not performed in our study. Deparaffinized and rehydrated samples were incubated for 15 min in a 3% H_2_O_2_ solution to inactivate endogenous peroxidase activity. A 2.5% horse normal serum from the ImmPRESS reagent kit anti-mouse IgG/anti-rabbit IgG (Vector Laboratories, Burlingame, CA, USA) was used for 30 min to block nonspecific binding sites. The samples were then incubated with the primary antibody at 4 °C overnight. After two washes with 0.1 M phosphate-buffered (pH 7.2) solution (PB), samples were incubated for 20 min with either the ImmPRESS VR Polymer HRP anti-rabbit IgG or anti-mouse IgG reagents (Table 1), except for the slides incubated with the anti-OMP antibody (raised in goat), which were incubated with a biotinylated anti-goat IgG for 1.5 h, and then incubated in avidin–biotin–peroxidase complex Vectastain reagent (ABC; Vector Laboratories, Burlingame, CA, USA). In all cases, successive 2 × 5 min PB washes were performed between steps. Finally, a 0.05% 3,3′-diaminobenzidine (DAB) chromogen solution and a 0.003% H_2_O_2_ solution, both in 0.2 M Tris–HCl buffer, were used. The DAB reagent develops into a brown precipitate in the presence of hydrogen peroxide solution, which enables visualization of the reaction. For better visualization of the DAB reaction product, the sections were not counterstained.

For all immunohistochemical procedures, the omission of the primary antibody was used as a negative control, and no labeling or non-specific background staining was observed for any negative control samples. As positive controls, we replicated the immunohistochemical procedure in previously unstained mouse or rabbit tissue obtained during previous experiments. These samples were known to express the proteins of interest, and the expected positive results were obtained in all cases

### 2.5. Lectin Histochemistry Protocol

#### 2.5.1. Lycopersicon Esculentum Agglutinin (LEA) Protocol

Biotinylated-conjugated LEA was employed (Table 1); 3% H_2_O_2_ and 2% BSA in PB were employed to inactivate endogenous peroxidase activity and to block nonspecific binding, respectively. Slides were then incubated overnight with LEA in a 0.5% BSA solution. Samples were washed 2 × 2 min in PB, and afterwards incubated for 1.5 h at room temperature with ABC reagent, before development with a DAB chromogen (same protocol as for the immunohistochemical labeling).

#### 2.5.2. Ulex Europaeus Agglutinin (UEA) Protocol

In this case, the slides were incubated for 1 h at room temperature in 0.5% BSA/UEA-I solution and then washed for 3 × 5 min in PB solution. The slides were then incubated overnight with an anti-UEA peroxidase-conjugated antibody. The next day, the samples were washed with a PB solution. DAB solution was added to visualize the reaction.

Controls were performed for both protocols, both without the addition of lectins and with the pre-absorption of lectins, by using an excess amount of the corresponding sugar.

### 2.6. Acquisition of Images

Images were captured with a Karl Zeiss Axiocam MRc5 digital camera coupled to a Zeiss Axiophot microscope. Brightness, contrast and balance levels were adjusted using Adobe Photoshop CS4 (Adobe Systems, San Jose, CA, USA). No specific characteristics within the images were altered, enhanced, moved or introduced.

## 3. Results

### 3.1. Dog Olfactory Bulb

#### 3.1.1. Macroscopic and Microscopic Anatomy

**Figure 1:**
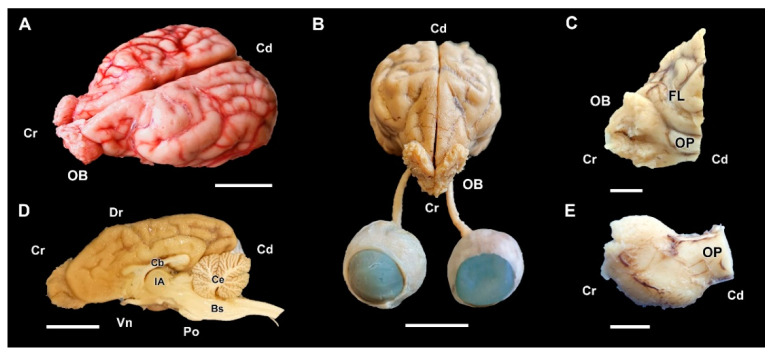
Macroscopic anatomy of the olfactory bulb (OB) of the dog. (**A**) Cranio-dorsolateral view of the encephalon. (**B**) Craniodorsal view showing the OB and its relationship to the optic nerves and eyes. (**C**) Lateral view of the left OB and the frontal lobe of the telencephalon (FL). (**D**) Medial view of the right hemiencephalon showing the rostral projection of the OB. (**E**) Dorsal view of the right OB. Bs, brainstem; Cb, callosal body; Ce, cerebellum; IA, interthalamic adhesion; OP, olfactory peduncle; Po, pons. Position terms: Cr, cranial; Cd, caudal; Dr, dorsal; Vn, ventral. (**A**): Unfixed brain; (**B**–**E**): Formalin fixation. Scale bars: (**A**,**B**,**D**): 2 cm; (**C**,**E**): 1 cm.

The major anatomical landmarks of the dog olfactory bulb are shown in Figure 1. Its main features correspond to the typical macrosmatic mammal. It occupies the rostral aspect of the brain, and in a medial view of the hemiencephalon it notoriously projects rostrally (Figure 1D). A thick olfactory peduncle connects the olfactory bulb to the rest of the structures of the rhinencephalon (Figure 1C,E).

**Figure 2:**
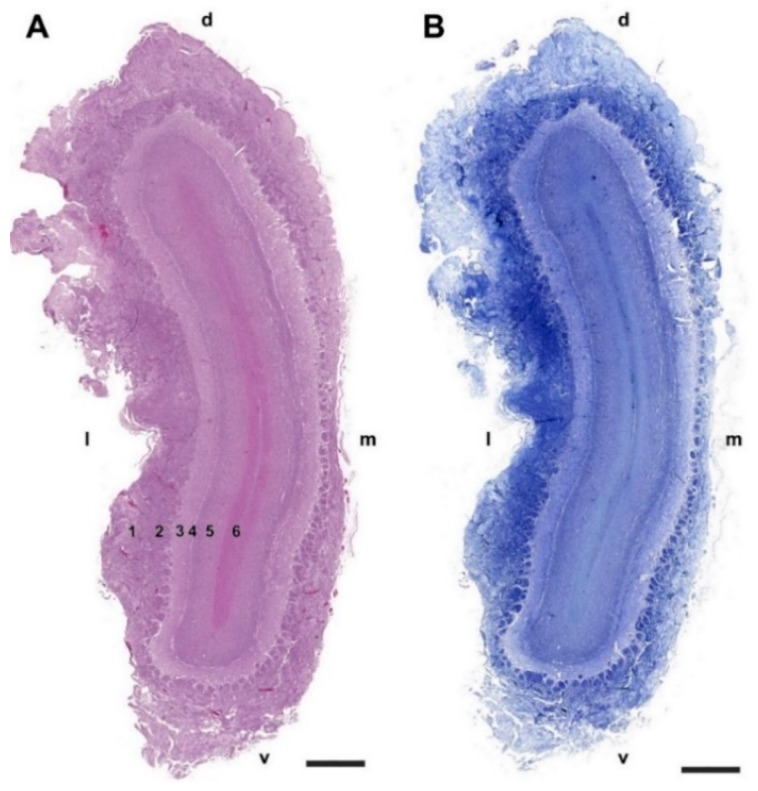
Transverse section of the olfactory bulb of the dog stained by hematoxylin–eosin (**A**) and Nissl staining (**B**). From superficial to deep the following layers are identified: (1) Olfactory nerve layer (NL); (2) Glomerular layer (GlL); (3) External plexiform layer (EPL); (4) Mitral cell layer (MCL); (5) Granular layer (GrL); (6) White matter. d, dorsal; l, lateral; m, medial; v, ventral. Scale bars: 2 mm.

Microscopically, transverse sections of the OB stained both with hematoxylin–eosin (Figure 2A) and Nissl (Figure 2B) clearly show lamination of the nerve tissue, which is evenly distributed along the entire perimeter of the bulb. However, the thickness of the layers is appreciably greater in the lateral half of the bulb than in the medial half.

**Figure 3:**
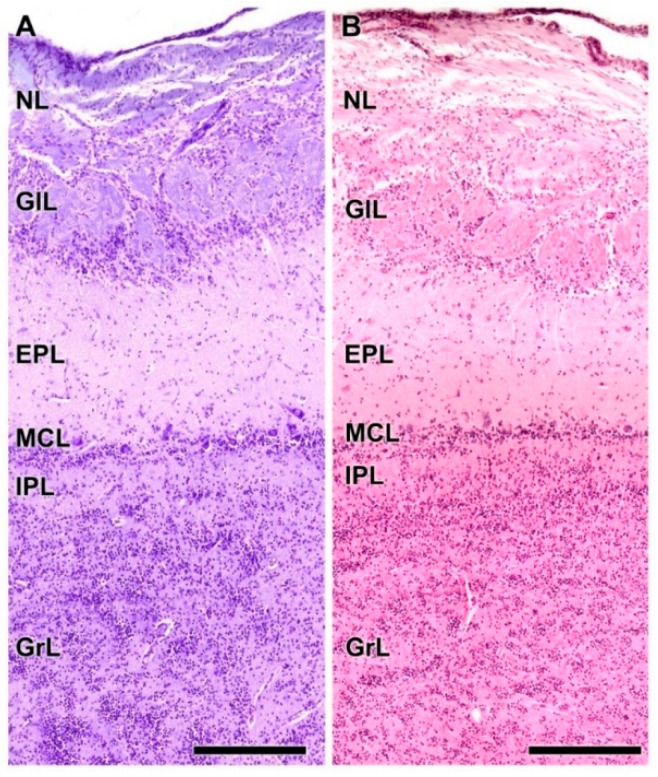
Histological study of the lamination of the olfactory bulb of the dog. Nissl (**A**) and hematoxylin–eosin staining (**B**) show the main layers of the MOB: olfactory nerve layer (NL), glomerular layer (GlL), external plexiform layer (EPL), mitral cell layer (MCL), internal plexiform layer (IPL) and granular layer (GrL). Scale bars: 250 µm.

As is typical of the MOB across mammalian species, seven layers are readily identified (Figure 3A,B). Moving from the superficial to deep planes, these layers include:

1. Nerve layer (NL): formed by the axons of the olfactory nerves that reach the OB.
2. Glomerular layer (GlL): comprised of glomeruli, spherical structures delimited by periglomerular (PG) cells. They correspond to the synapses of olfactory axons with the dendrites of mitral cells, which are the second neurons in the olfactory pathways.
3. External plexiform layer (EPL): a nerve plexus mainly occupied by the dendrites of mitral cells.
4. Mitral cell layer (MCL): a linear layer containing the somas of mitral cells.
5. Internal plexiform layer (IPL): a thin band of white matter interposed between the mitral and granular layers.
6. Granular layer (GrL): composed of large clusters of granule cells, which act as inhibitory neurons in the OB neural circuit.
7. White matter (WM): formed by the projecting axons of the OB (Figure 2A,B).

**Figure 4:**
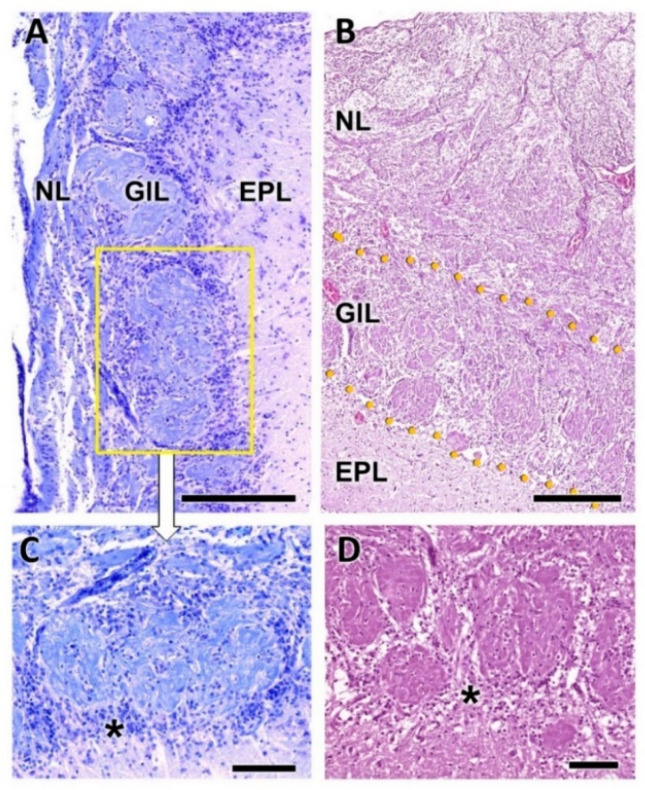
Histological study of the superficial layers of the olfactory bulb of the dog. (**A**) Nissl staining of the nerve layer (NL), glomerular layer (GlL) and external plexiform layer (EPL). (**B**) Hematoxylin–eosin staining. The GlL is outlined by yellow dots. (**C**) Enlargement of the box in (**A**), showing the periglomerular cells (*) concentrated in the border between the glomeruli and the EPL. Nissl staining. (**D**) Similar image of the glomerular layer stained by hematoxylin–eosin. Scale bars: (**A**,**B**): 250 µm; (**C**,**D**): 100 µm.

The olfactory nerve layer (Figure 4A,B) shows thick bundles of unmyelinated fibers that spread all over the surface of the OB. It is appreciable how particular bundles penetrate into the underlying layer of glomerulus (Figure 4C,D). The glomerular layer is comprised of large, spherical glomeruli, clearly delineated by the periglomerular cells, especially in their innermost part (Figure 4B). Rarely, the glomerular layer contains large neurons.

**Figure 5:**
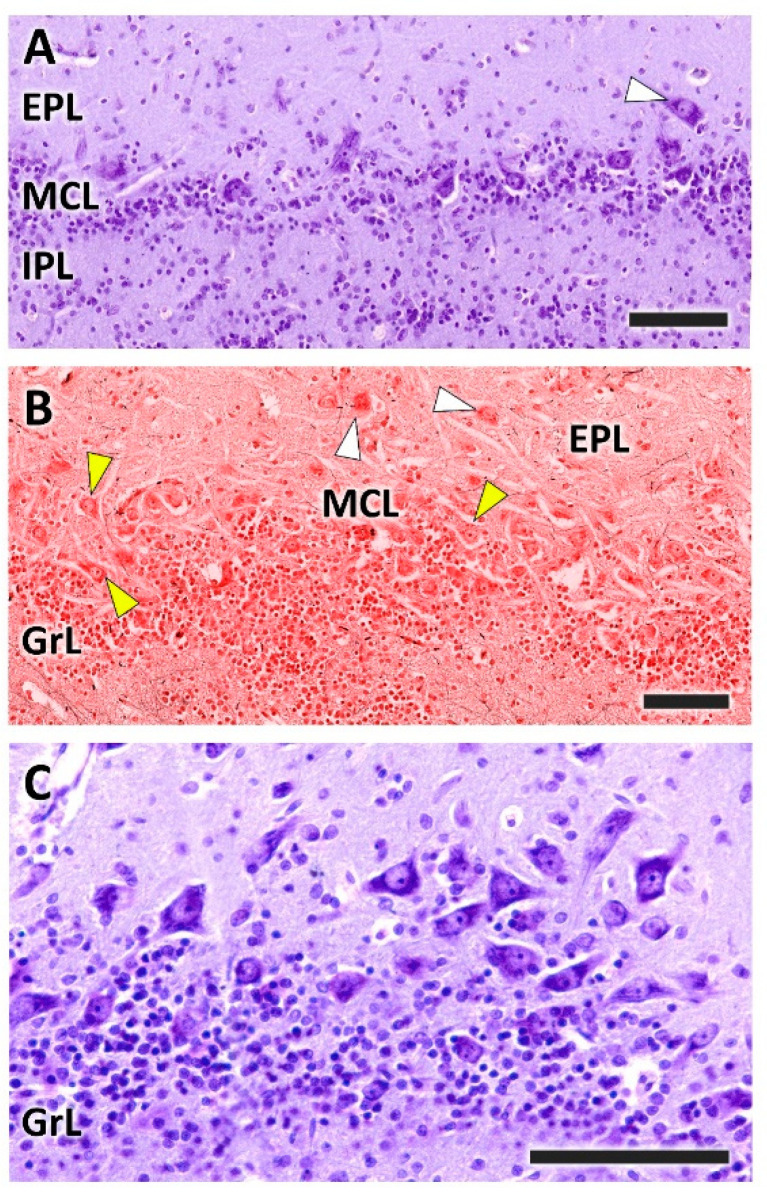
Histological study of the mitral cell layer of the olfactory bulb of the dog. (**A**,**B**) Nissl and Tolivia staining, respectively, of the mitral cell layer (MCL) showing typical mitral cells (yellow arrowheads) and deep tufted cells (white arrowheads) scattered along the boundaries between the external (EPL) and internal plexiform layers (IPL). (**B**,**C**) Sagittal section of the OB reveals a higher density of mitral cells, which are mitered or triangular in shape. Tolivia and Nissl staining, respectively. Scale bars: (**A**,**B**): 250 µm; (**C**): 100 µm.

The outer plexiform layer contains some tufted cells, mainly in a deep plane close to the mitral cell layer (Figure 5A,B). The mitral cell layer consists of an irregular alignment of mitral cells, which are intermingled with granular cells. In a sagittal section of the OB, a higher concentration of mitral cells is observed (Figure 5B,C). This allows one to appreciate the different sizes and morphologies—triangular, mitral, elliptical—that mitral cells can present.

**Figure 6:**
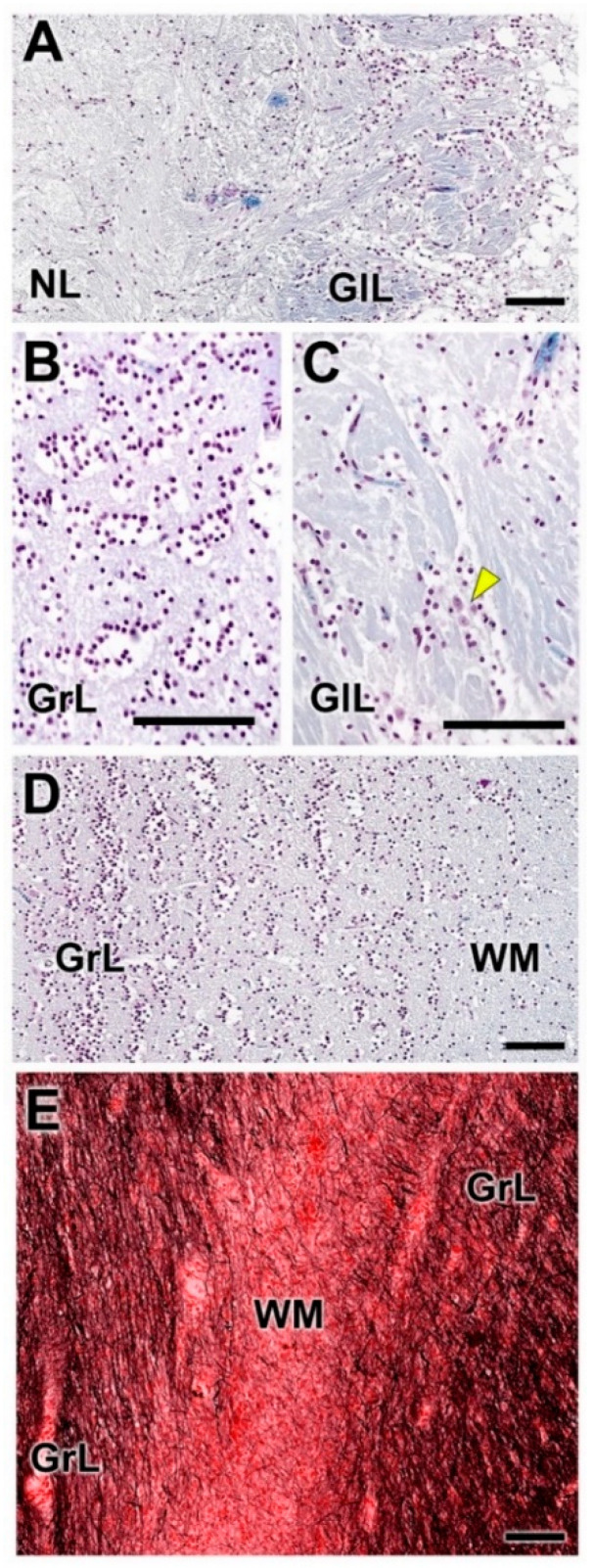
Histological study of the dog olfactory bulb stained with Gallego’s trichrome and Tolivia staining. (**A**,**C**) Nerve and glomerular layers (NL and GlL, respectively). This staining allows good definition of the periglomerular cells. At higher magnification (**C**), scattered neurons with large somas are also found (arrowhead). (**B**,**D**) The granular layer (GrL) is formed by clusters of granule cells. (**E**). The deep area of the OB contains the white matter (WM). Tolivia staining shows a higher density of myelinic fibres in the GrL than in the WM. Scale bars: 100 µm.

Gallego’s trichrome staining highlights the arrangement of periglomerular cells (Figure 6A,C) and the granular cells in concentric alignments (Figure 6B,D). In a deeper plane, the white matter can be seen, which, as can be determined with Tolivia staining, is less rich in myelin fibers than the granule cell layer (Figure 6E).

#### 3.1.2. Immunohistochemical and Lectin Histochemical Study

**Figure 7:**
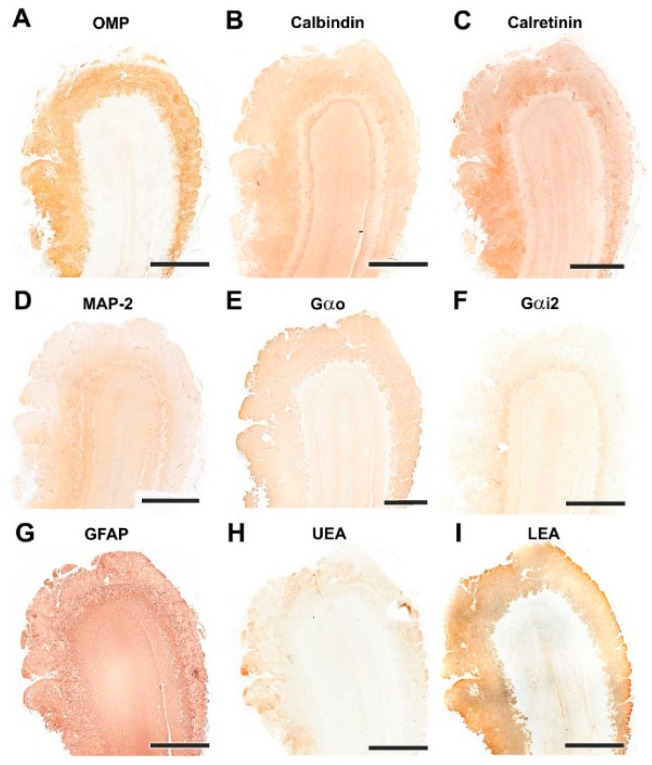
Immunohistochemical and lectin-histochemical study of the OB of the dog. (**A**) Anti-Olfactory marker protein (OMP). (**B**) Anti-Calbindin. (**C**) Anti-Calretinin. (**D**) Anti-MAP-2. (**E**) Anti-Gao. (**F**) Anti-Gai2. (**G**) Anti-GFAP. (**H**) UEA histochemical labeling. (**I**) LEA histochemical labeling. Scale bars: 2 mm.

**Figure 8:**
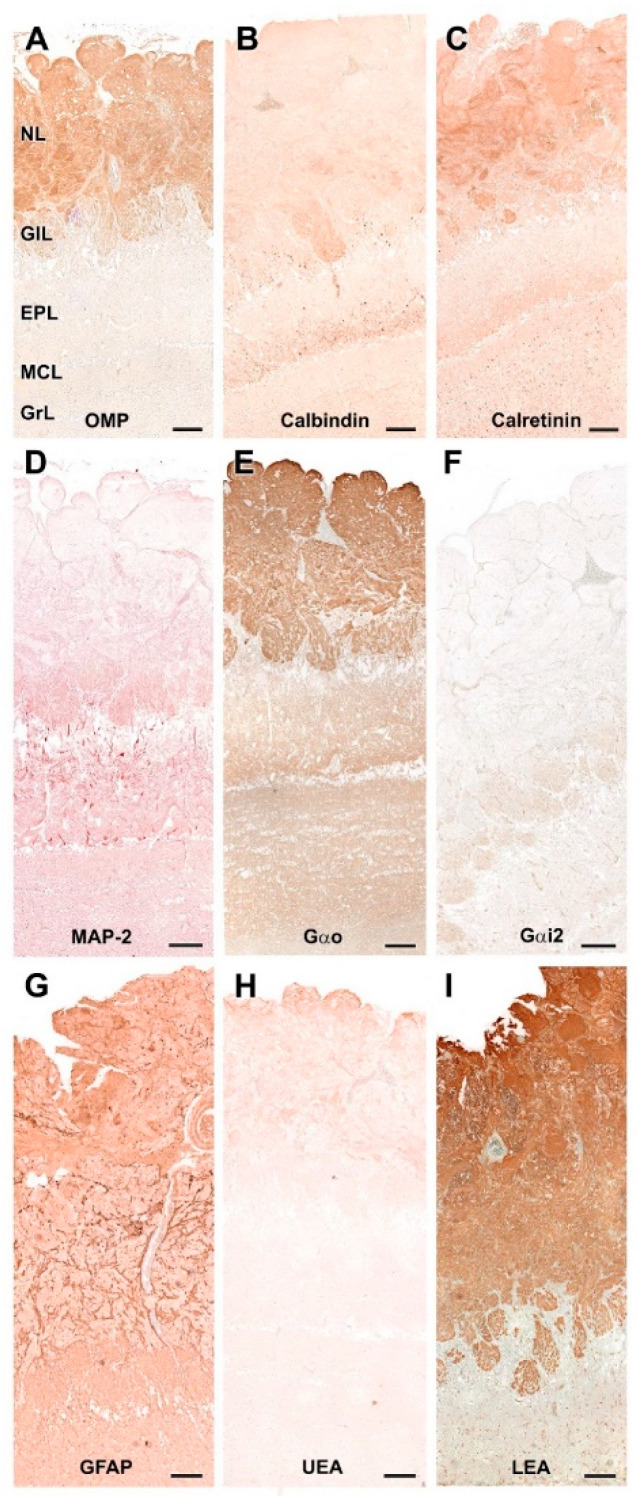
Immunohistochemical and lectin-histochemical study of the OB of the dog. (**A**) Anti-Olfactory marker protein (OMP). (**B**) Anti-Calbindin. (**C**) Anti-Calretinin. (**D**) Anti-MAP-2. (**E**) Anti-Gao. (**F**) Anti-Gai2. (**G**) Anti-GFAP. (**H**) UEA histochemical labeling. (**I**) LEA histochemical labeling. NL, Nerve layer. GIL, Glomerular layer. EPL, External plexiform layer. MCL, Mitral cell layer. IPL, Internal plexiform layer; GrL, Granular layer. Scale bars: 100 mm.

Each of the antibodies and lectins used had a characteristic labeling pattern. The outermost superficial layers, nerve and glomerular, were labeled in a specific manner by both anti-OMP (Figure 7A), a marker specific for mature olfactory neurons, and by the lectin LEA (Figure 7I), specific for *N*-acetyl-glucosamine. The glomerular morphology appears more sharply demarcated with LEA than with anti-OMP (Figure 8A,I). Anti-Gao, specific for the G protein ao subunit, produces equally intense labeling in the superficial layers, but this protein, although with much less intensity, is also expressed in the deep layers of the bulb (Figure 7E and Figure 8E). In contrast, the other G-protein subunit studied, ai2, is only faintly expressed in the glomerular layer (Figure 7F and Figure 8F).

The calcium binding proteins calbindin (CB) and calretinin (CR) are expressed in both cases in all layers of the bulb, although with slightly different patterns (Figure 7B,C). In the case of CB, the glomerular, mitral plexiform and granular layers are immunostained (Figure 7B). At the cellular level, the periglomerular cells as well as the first dendritic arborizations of the mitral cell layer show CB immunopositivity (Figure 8B). In the case of CR, the two superficial layers, nerve and glomerular, show immunopositivity. At the cellular level, anti-CR produces immunopositivity in both periglomerular and granular cells (Figure 8C).

The dendritic projections of the mitral cells were labeled with antibodies against microtubule-associated protein 2 (MAP-2) (Figure 7D and Figure 8D). Additionally, the glomerular layer showed intense, diffuse immunolabeling. Astrocytes and ensheathing cells were identified throughout the olfactory bulb by an antibody against glial fibrillary acidic protein (GFAP) (Figure 7G), with labeling being concentrated in the superficial layers, especially in the nerve layer, where ensheathing cell processes accompany the olfactory axon terminals (Figure 8G). Finally, UEA, an L-fucose specific lectin, produced a negative pattern in all layers of the olfactory bulb (Figure 7H and Figure 8H).

### 3.2. Wolf Olfactory Bulb

#### 3.2.1. Macroscopic and Microscopic Anatomy

**Figure 9:**
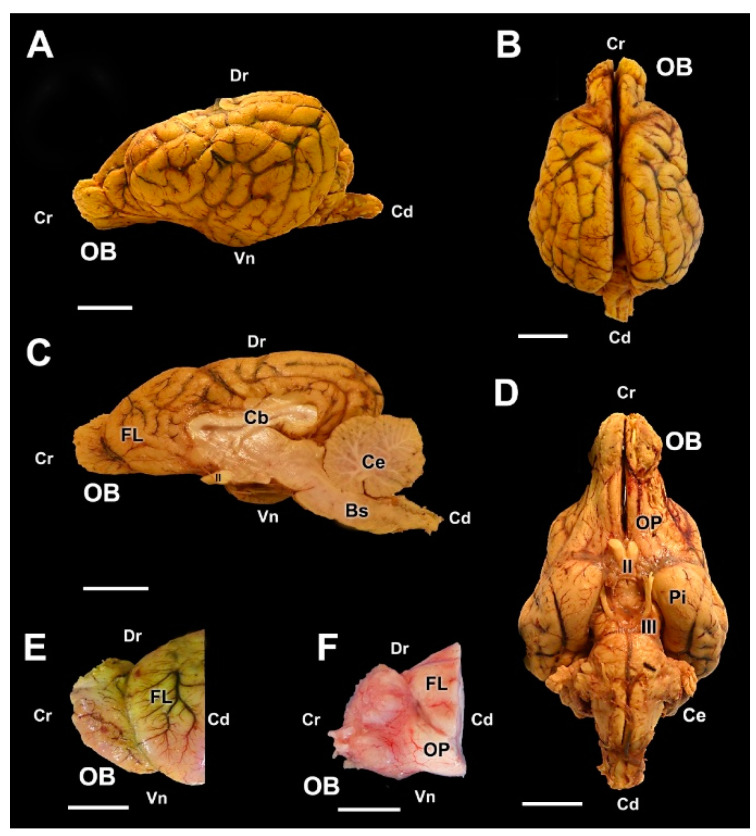
Macroscopic anatomy of the olfactory bulb (OB) of the wolf. (**A**,**B**) Lateral and dorsal view of the encephalon, respectively. (**C**) Medial view of the right hemiencephalon. (**D**) Ventral view of the encephalon. (**E**) Medial view of the right OB and the frontal lobe (FL) of the telencephalon. (**F**) Lateral view of the left OB and the frontal lobe of the telencephalon. (II) Optical nerve; (III) Oculomotor nerve; Bs, Brainstem; Cb, Callosal body; Ce, Cerebelum; OP, olfactory peduncle; Pi, Piriform lobe. Position terms: Cr, Cranel; Cd, Caudal; Dr, dorsal; Vn, Ventral. (**A**–**E**): Bouin’s liquid fixation. (**F**): Unfixed sample. Scale bars: (**A**–**D)**: 2 cm; (**E**,**F**): 1 cm.

The macroscopic study of the wolf’s brain shows well-developed olfactory bulbs, very prominent both in the lateral view of the brain (Figure 9A) and in the medial view of the hemiencephalon (Figure 9C,E). Also remarkable are the well-developed rhinencephalon, which features a broad olfactory pendunculi (Figure 9F) and piriform lobes of considerable width and convexity (Figure 9D).

**Figure 10:**
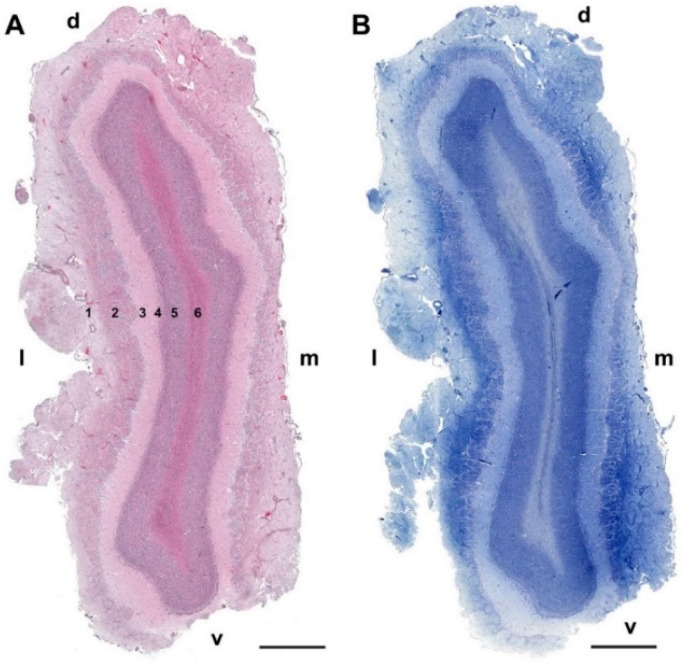
Transverse section of the olfactory bulb of the wolf stained by hematoxylin–eosin (**A**) and Nissl staining (**B**). From superficial to deep, the following layers are identified: (1) Olfactory nerve layer (NL); (2) Glomerular layer (GlL); (3) External plexiform layer (EPL); (4) Mitral cell layer (ML); (5) Granular layer (GrL); (6) White matter: d, dorsal; l, lateral; m, medial; v, ventral. Scale bars: 2 mm.

**Figure 11:**
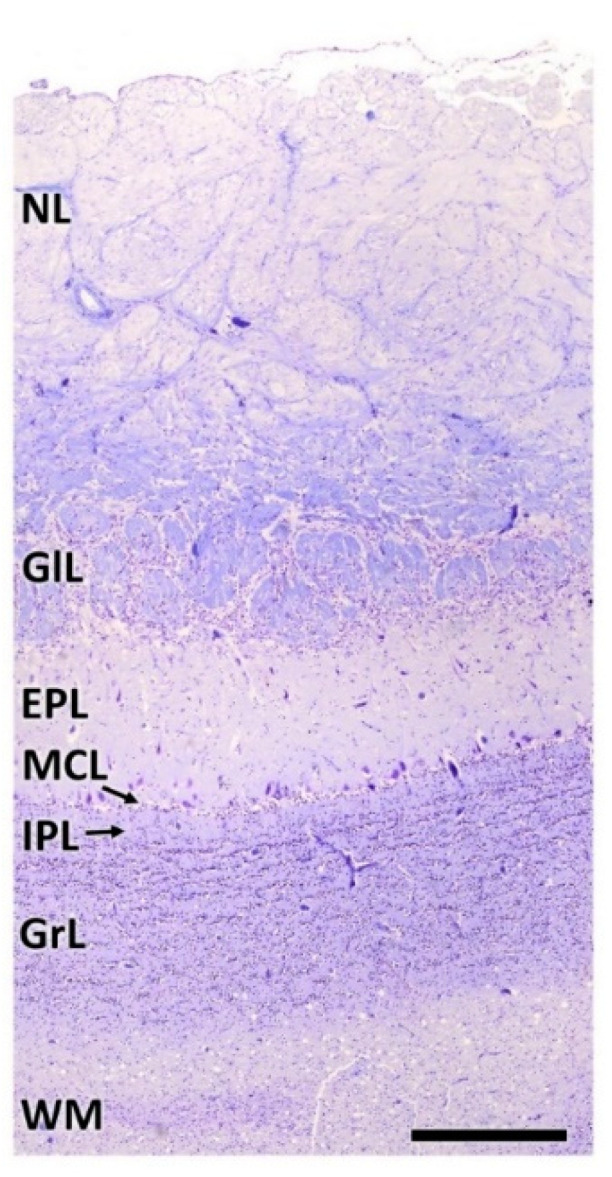
Lamination of the wolf OB. NL, Nerve layer. GlL, Glomerular layer. EPL, External plexiform layer. MCL, Mitral cell layer; IPL, Internal plexiform layer; GrL, Granular layer; WM, White matter. Nissl staining. Scale bar: 1 mm.

Histological examination of the OB shows the typical mammalian lamination (Figure 10 and Figure 11) with a degree of differentiation and development of the layers characteristic of macrosmatic species. The nerve and glomerular layers are particularly thick.

**Figure 12:**
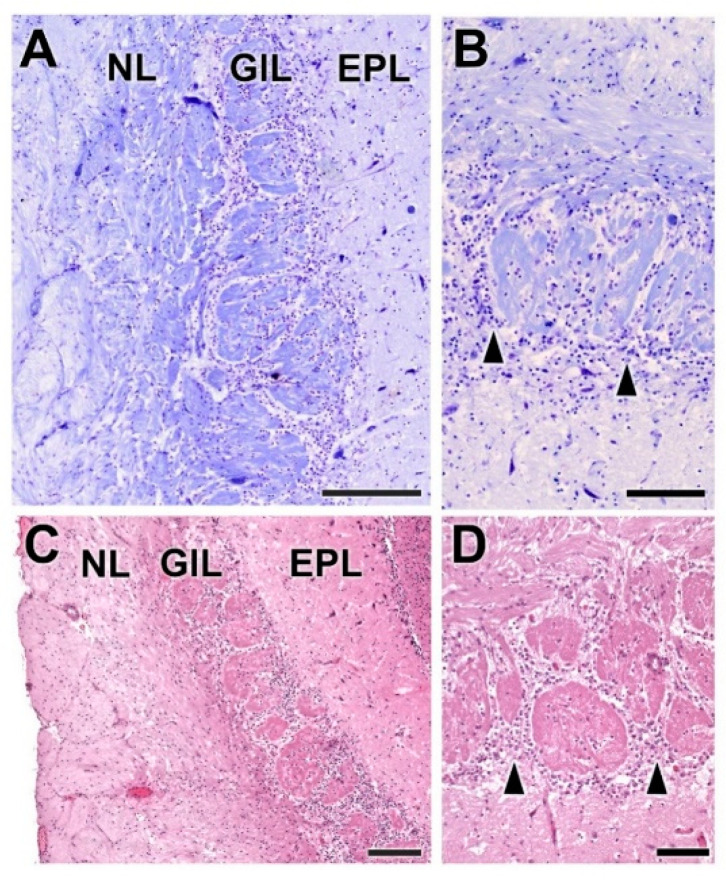
Histological study of the superficial layers of the olfactory bulb of the wolf. (**A**,**B**) Nissl staining of the nerve (NL), glomerular (GlL) and external plexiform (EPL) layers. The arrowheads indicate the presence of periglomerular cells. (**C**,**D**) Hematoxylin–eosin staining. The periglomerular cells are mainly located in the border between the glomeruli and the EPL (arrowheads). Scale bars: (**A**): 250 µm; (**B**): 125 µm; (**C**,**D**): 100 µm.

**Figure 13:**
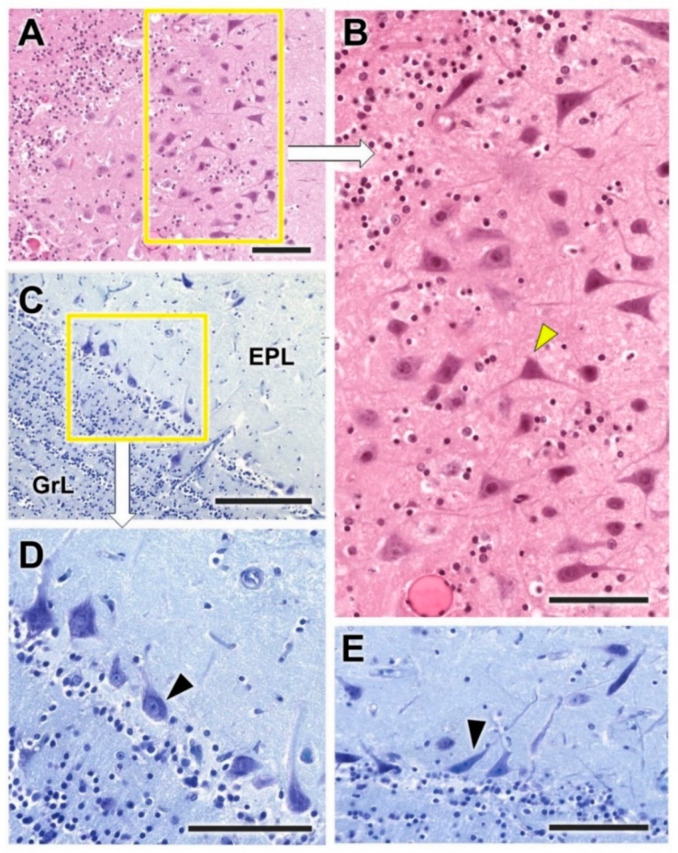
Histological study of the mitral cell layer of the olfactory bulb of the wolf. (**A**) Hematoxylin–eosin staining (box enlarged in (**B**)) of a sagittal section of the mitral cell layer (MCL) showing typical mitral cells (arrowheads) with big soma and very well-defined dendritic projections. (**C**–**E**) Nissl staining of transverse sections of the MCL showing the diverse morphology of the mitral cell somas. Scale bars: 100 µm.

Both the nerve and the glomerular layer were characterized by Nissl and hematoxylin–eosin staining (Figure 12). The GlL consisted of 2–4 layers of glomeruli, surrounded by a high density of periglomerular cells (Figure 12D). The mitral cell layer showed, in both transverse (Figure 13C,E) and sagittal sections (Figure 13A,B), numerous mitral cells (arrowheads) featuring either big triangular or miter-shaped somas and very well-defined multidendritic projections.

#### 3.2.2. Immunohistochemical and Lectin Histochemical Study

**Figure 14:**
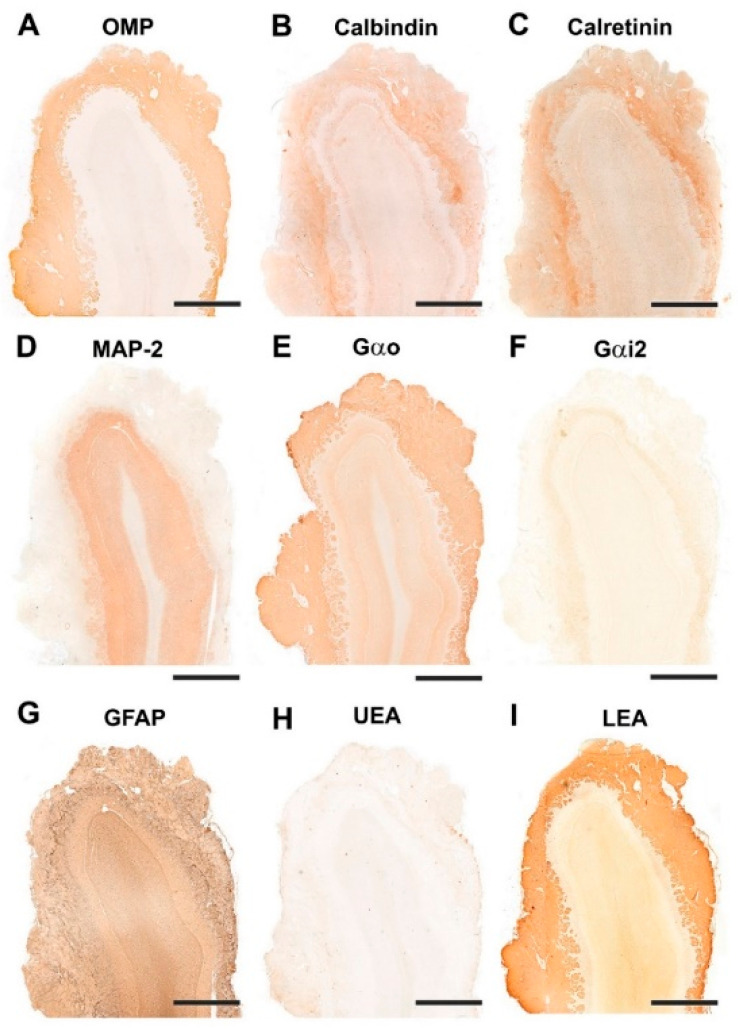
Immunohistochemical and lectin-histochemical study of the OB of the wolf. (**A**) Anti-Olfactory marker protein (OMP); (**B**) Anti-Calbindin; (**C**) Anti-Calretinin; (**D**) Anti-MAP-2; (**E**) Anti-Gao; (**F**) Anti-Gai2; (**G**) Anti-GFAP; (**H**) UEA histochemical labeling; (**I**) LEA histochemical labeling. Scale bars: 2 mm.

**Figure 15:**
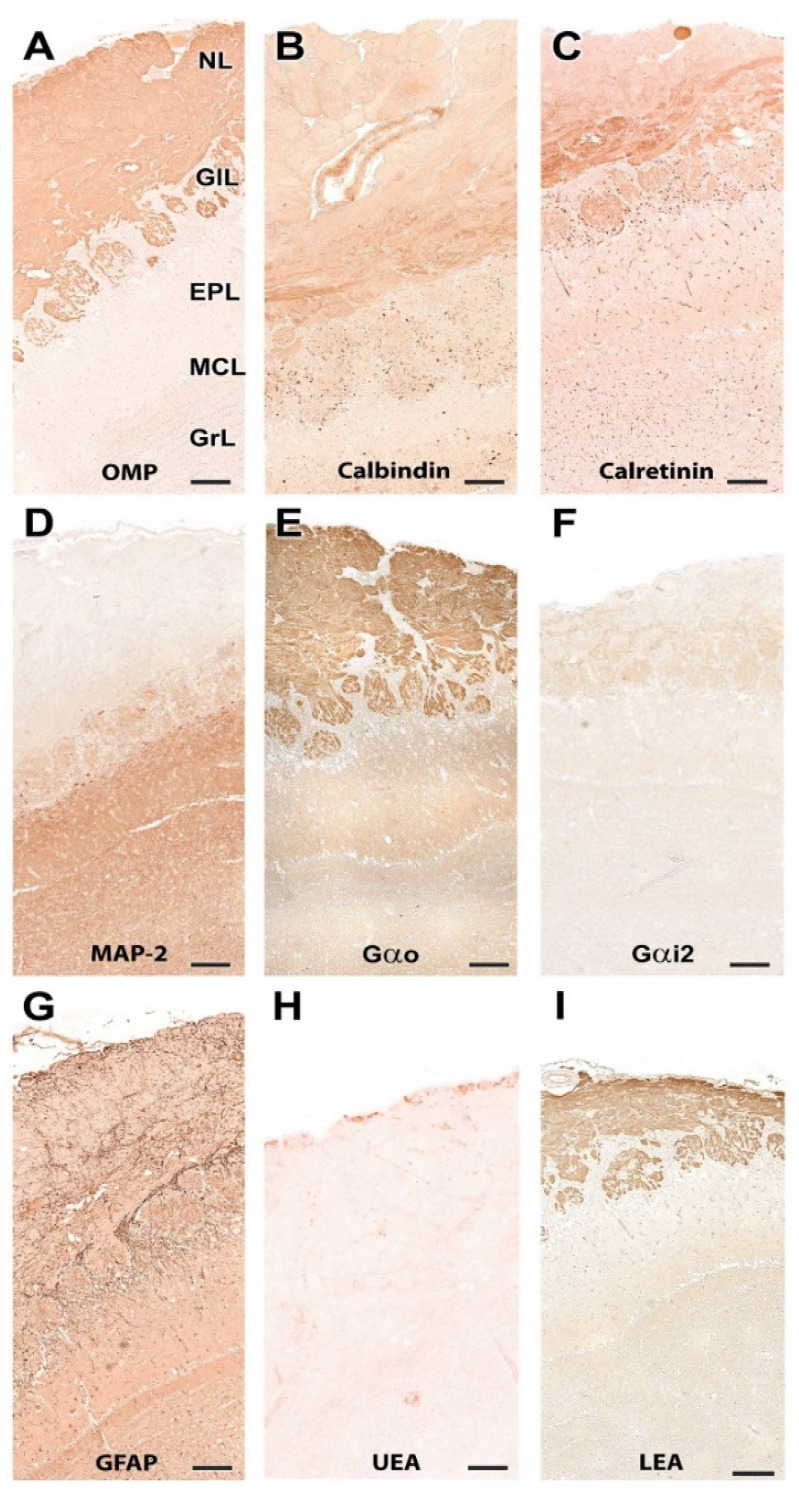
Immunohistochemical and lectin-histochemical study of the OB of the wolf. (**A**) Anti-Olfactory marker protein (OMP); (**B**) Anti-Calbindin; (**C**) Anti-Calretinin; (**D**) Anti-MAP-2; (**E**) Anti-Gao; (**F**) Anti-Gai2; (**G**) Anti-GFAP; (**H**) UEA histochemical labeling; (**I**) LEA histochemical labeling. NL, Nerve layer; GlL, Glomerular layer; EPL, External plexiform layer; MCL, Mitral cell layer; GrL, Granular layer. Scale bars: 100 mm.

Immunohistochemical and lectin staining (Figure 14 and Figure 15) show in the wolf a similar labeling pattern to that described in the dog, with a very clear definition of the layers. OMP (Figure 14A and Figure 15A) and LEA (Figure 14I and Figure 15I) are specific markers for the superficial layers of the OB, both nerve and glomerular layers, but do not stain the deep layers. This implies that the glomerular layer labeling derives exclusively from axonal afferences originating from the olfactory nerves. Gao (Figure 14E and Figure 15E) is expressed in both the superficial layers and in the deep layers, mainly at the level of the mitral cell layer, which contributes to the labeling of the glomerular layer. Gai2 (Figure 14F and Figure 15F) is immunonegative for the whole OB. In contrast to LEA, UEA lectin, as happens in the dog, does not stain the OB (Figure 14H and Figure 15H).

The calcium-binding proteins CB (Figure 14B and Figure 15B) and CR (Figure 14C and Figure 15C), show a broad labeling pattern comprising both fibers and cellular elements. Both markers are highly expressed in periglomerular cells. Additionally, anti-CR produces intense labeling in granular cells. In addition to the surface layers, anti-CB stains the mitral cell layer intensely, but does not show immunoreactivity in the mitral cells themselves.

Anti-MAP-2 (Figure 14D and Figure 15D) strongly stains the glomerular, external plexiform, mitral and granular layers. Not staining the olfactory nerve layer implies that glomerular immunopositivity corresponds to the dendrites of the deep cells of the OB, the most abundant being the mitral and granular cells. Finally, anti-GFAP (Figure 14G) reveals the dense network shaped by astrocytes and ensheathing cell prolongations. They are mostly concentrated in the superficial layers, where the processes distinctly delimit the fascicles of the olfactory nerves and glomerular formations (Figure 15G).

### 3.3. Fox Olfactory Bulb

#### 3.3.1. Macroscopic and Microscopic Anatomy

**Figure 16:**
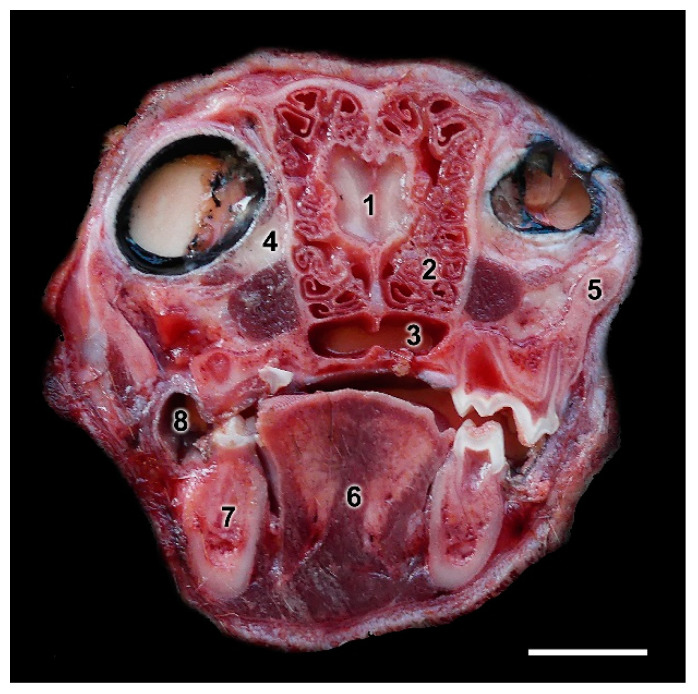
Transverse macroscopic section of the head of the fox at the level of the olfactory bulb (OB) showing its main topographic relationships. (1) Olfactory bulb; (2) Ethmoidal concha; (3) Choanas; (4) Zygomatic gland; (5) Zygomatic arch; (6) Tongue; (7) Mandible; (8) Buccolingual recess. Scale bar: 2 cm.

**Figure 17:**
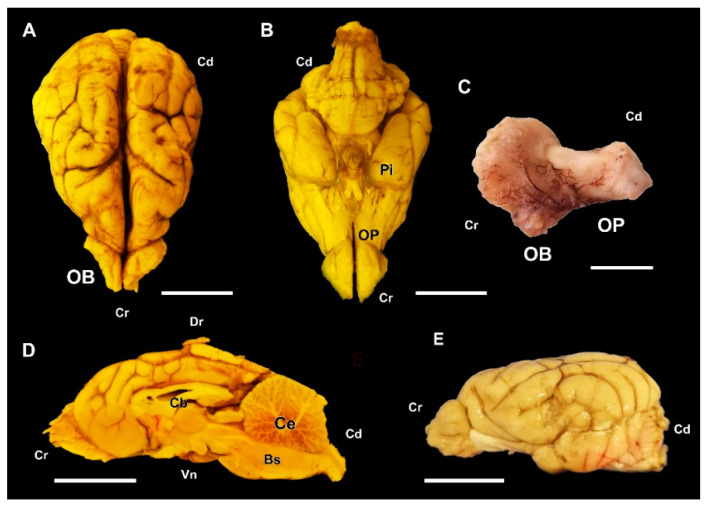
Macroscopic anatomy of the olfactory bulb (OB) of the fox. (**A**,**B**) Dorsal and ventral view of the encephalon, respectively. (**C**) Medial view of the right OB after removing the frontal lobe. (**D**) Medial view of the right hemiencephalon. (**E**) Lateral view of the encephalon. Bs, Brainstem; Cb, Callosal body; Ce, Cerebellum; OP, olfactory peduncle; Pi, Piriform lobe. Position terms: Cr, Cranial; Cd, Caudal; Dr, dorsal; Vn, Ventral. (**A**,**B**,**D**): Bouin’s liquid fixation. (**C**,**E**): Formalin fixation. Scale bars: (**A**,**B**,**D**,**E**): 2 cm; (**C**): 0.5 cm.

A transverse section of the head at the level of the ethmoidal fossa (Figure 16), just between both eyes, shows the topographical relationships of the OB. The olfactory bulb is completely surrounded by the ethmoidal concha, which contains the olfactory neuroepithelium that projects the olfactory axons to the olfactory nerve layer of the olfactory bulb. Relative to brain size, the fox presents well-developed and kidney-shaped olfactory bulbs (Figure 17). In a ventral view of the brain, the rest of the structures of the rhinencephalon are visible: the olfactory peduncle, olfactory tubercle and the piriform lobe (Figure 17B).

**Figure 18:**
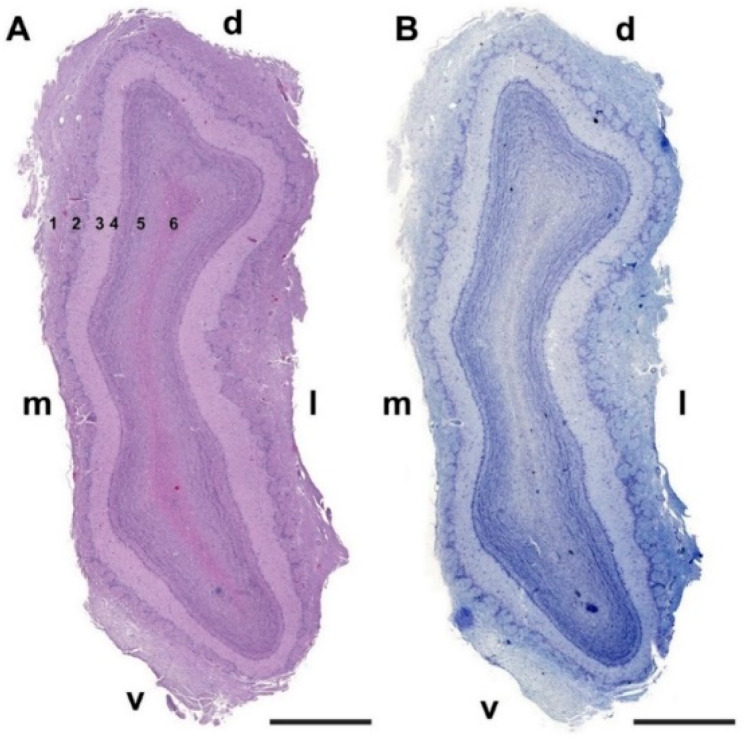
Transverse section of the olfactory bulb of the red fox stained by hematoxylin–eosin (**A**) and Nissl staining (**B**) From superficial to deep the following layers are identified: (1) Olfactory nerve layer (NL); (2) Glomerular layer (GlL); (3) External plexiform layer (EPL); (4) Mitral cell layer (ML); (5) Granular layer (GrL); (6) White matter: d, dorsal; l, lateral; m, medial; v, ventral. Scale bars: 2 mm.

Histological study of the fox OB shows a clear lamination (Figure 18) with a degree of differentiation and development of the layers typical of macrosmatic species. Both the nerve and glomerular layers are particularly thick in the lateral half of the OB.

**Figure 19:**
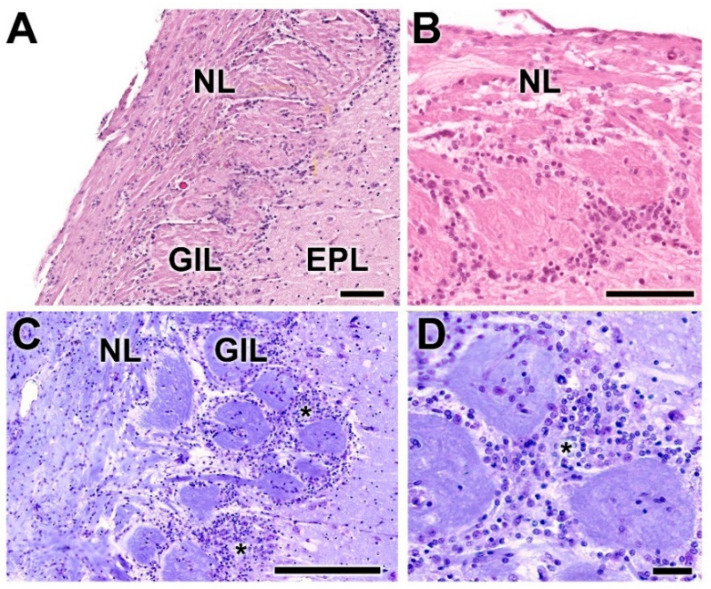
Histological study of the superficial layers of the olfactory bulb of the fox. (**A**,**B**) Hematoxylin–eosin staining of the nerve layer (NL), glomerular layer (GlL) and external plexiform layer (EPL). The asterisk indicates the presence of periglomerular cells. (**C**,**D**) Nissl staining. There is a high density of periglomerular cells in the border between the glomeruli and the EPL (asterisks). Scale bars: (**A**,**B**,**D**): 100 µm; (**C**): 250 µm.

**Figure 20:**
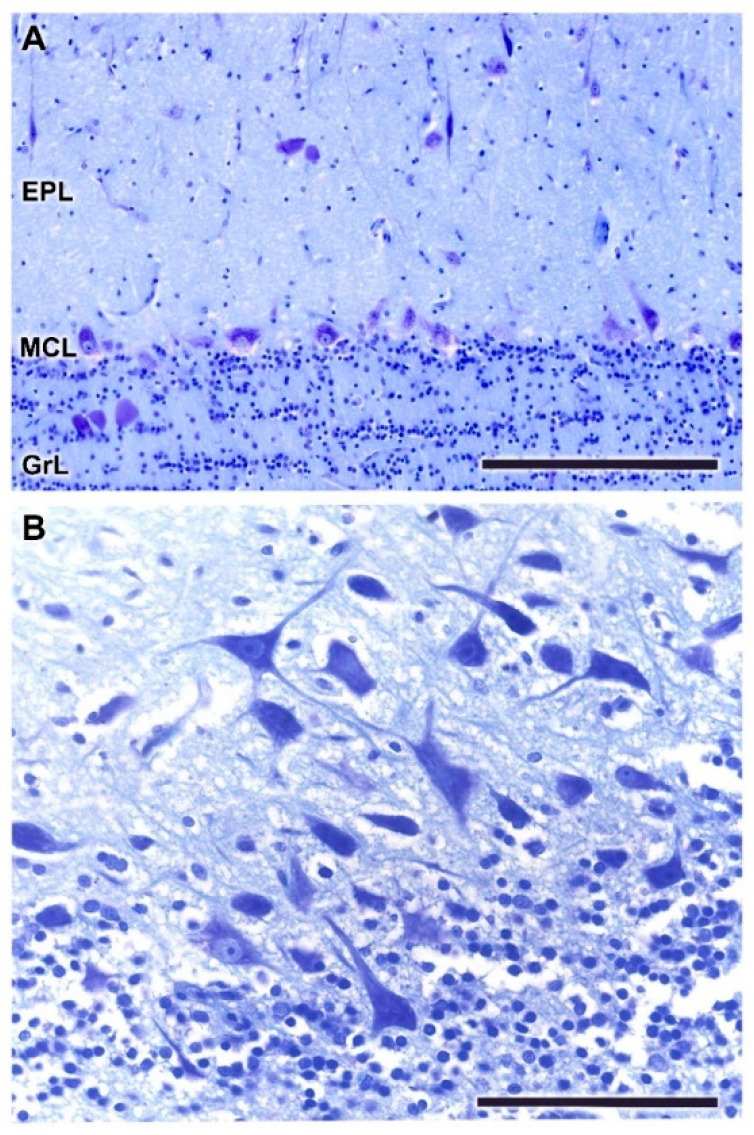
Histological study of the mitral cell layer of the olfactory bulb of the fox. (**A**) Nissl staining of a transverse section of the olfactory bulb showing mitral cell somas aligned along the MCL. (**B**) A sagittal section of the mitral cell layer (MCL) stained with Nissl staining shows the typical mitral cells characterized by their big soma and well-defined dendritic projections. Scale bars: (**A**): 250 µm. (**B**): 100 µm.

**Figure 21:**
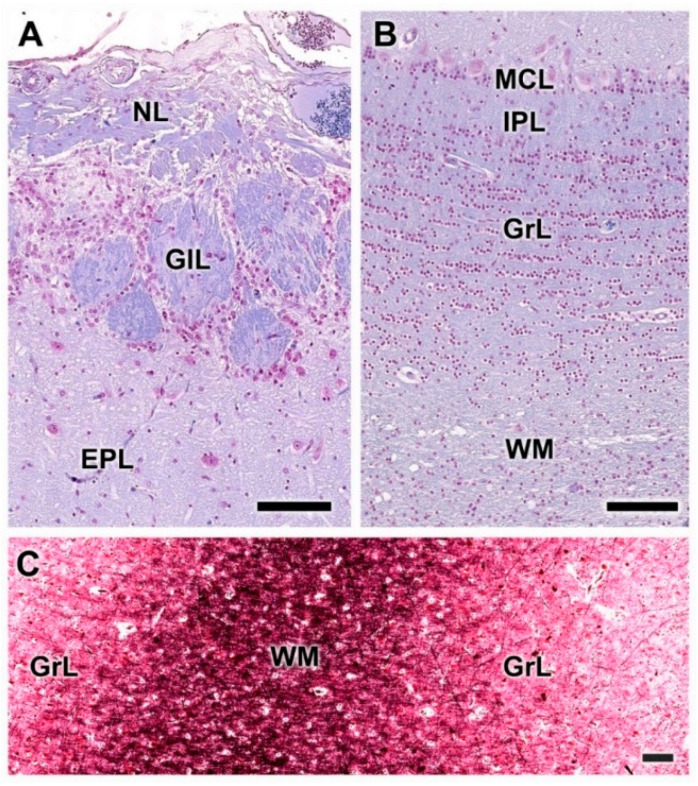
Histological study of the olfactory bulb of the fox stained with Gallego’s trichrome. (**A**,**B**) Superficial and deep layers, respectively. The glomeruli are wholly surrounded by periglomerular cells. The granular layer (GrL) is formed by aligned clusters of granule cells. (**C**) Tolivia staining shows a high density of myelinic fibers in white matter (WM). Scale bars: 100 µm.

Nissl and hematoxylin–eosin staining (Figure 19 and Figure 20) were employed to histologically characterize the OB of the fox. The GlL consisted of 2–3 layers of glomeruli, smaller than those described in the wolf, but likewise surrounded by a high density of periglomerular cells (Figure 19C,D and Figure 21A). The mitral cell layer showed numerous mitral cells in both transverse (Figure 20A and Figure 21B) and sagittal (Figure 20B) Nissl-stained sections, similar in shape and development of their processes to those found in the wolf. Deep to the mitral cell layer there was a thick band corresponding to the inner plexiform layer (Figure 21B), several concentric linear clusters of granular cells (Figure 21B) and a broad layer of white matter, mostly consisting of amyelinic fibers (Figure 21C).

#### 3.3.2. Immunohistochemical and Lectin Histochemical Study

**Figure 22:**
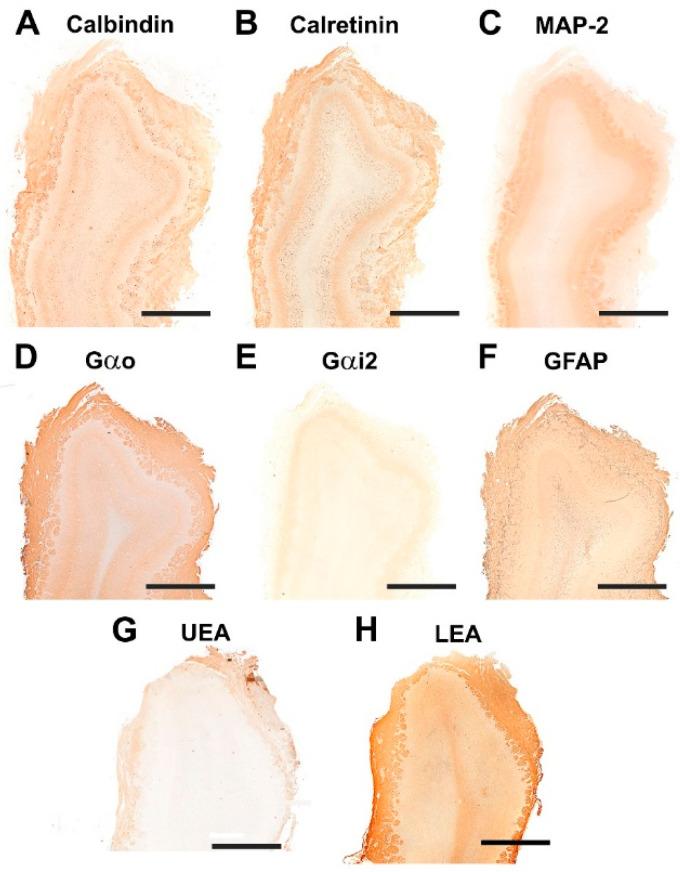
Immunohistochemical and lectin-histochemical study of the OB of the fox. (**A**) Anti-Calbindin; (**B**) Anti-Calretinin; (**C**) Anti-MAP-2; (**D**) Anti-Gao; (**E**) Anti-Gai2; (**F**) Anti-GFAP; (**G**) UEA histochemical labeling; (**H**) LEA histochemical labeling. Scale bars: 2 mm.

**Figure 23:**
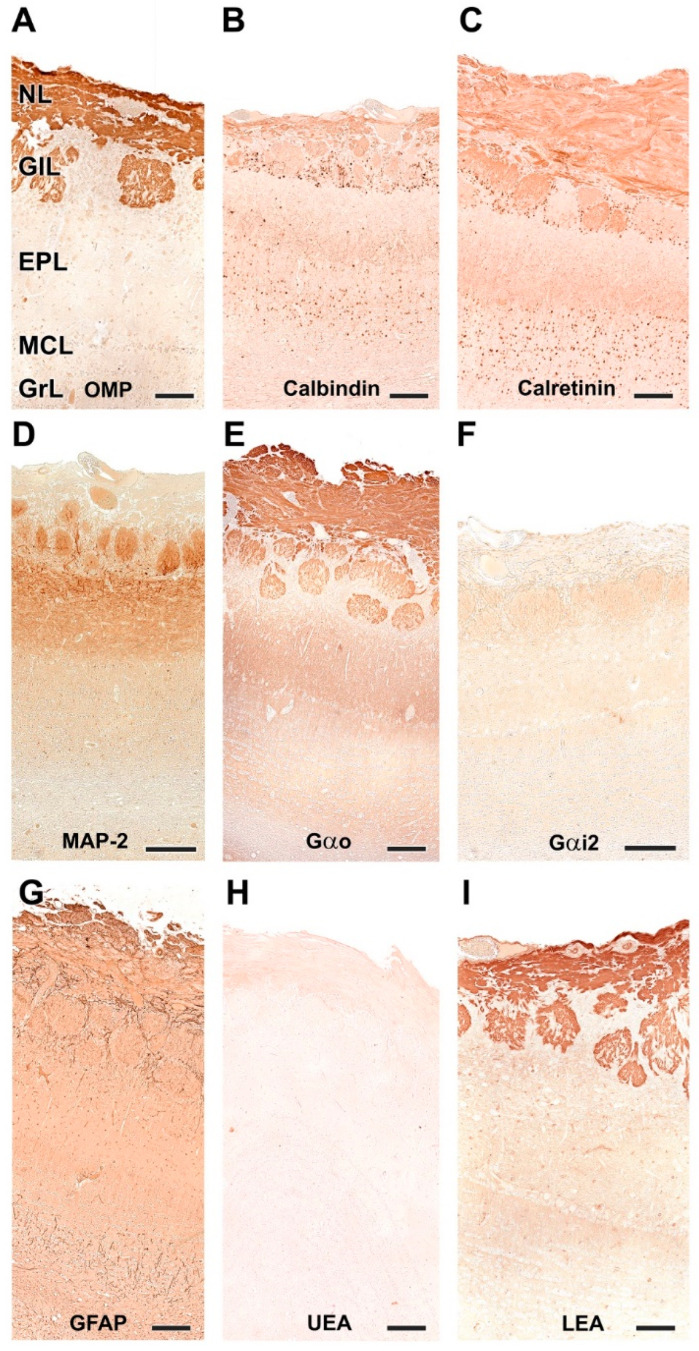
Immunohistochemical and lectin-histochemical study of the olfactory bulb of the fox. (**A**) Anti-Olfactory marker protein (OMP); (**B**) Anti-Calbindin; (**C**) Anti-Calretinin; (**D**) Anti-MAP-2; (**E**) Anti-Gao; (**F**) Anti-Gai2; (**G**) Anti-GFAP; (**H**) UEA histochemical labeling; (**I**) LEA histochemical labeling. NL, Nerve layer; GlL, Glomerular layer; EPL, External plexiform layer; MCL, Mitral cell layer; GrL, Granular layer. Scale bars: 100 mm.

The pattern of immunohistochemical and histochemical lectin labeling in the fox OB is similar to that observed in dog and wolf, with a high specificity towards the superficial, nervous and glomerular layers by LEA (Figure 22H and Figure 23I) and anti-OMP (Figure 23A). Anti-Gao (Figure 22D and Figure 23E) and anti-Gai2 (Figure 22E and Figure 23F) produced complementary immunolabeling for both markers, immunopositive for the former and immunonegative for the latter. CB (Figure 22A and Figure 23B) and CR (Figure 22B and Figure 23C) are expressed following a laminar distribution, comparable to that observed for dog and wolf. For both markers, in the fox, as in the dog and wolf, the intensity of marking in the glomeruli is variable within the same specimen, with no organized pattern. At the cellular level, both markers strongly stain the periglomerular and granular cells (Figure 23B,C).

Anti-MAP-2 (Figure 22C and Figure 23D) produces in the glomerular and external plexiform layer strong labeling of the dendritic arbors originating from mitral cells, similar to that observed in the wolf and more intense than that described in the dog. This result corresponds to the greater morphological development observed with histological staining of mitral cells in wild canids. Anti-GFAP (Figure 22F and Figure 23G) provides evidence of glial cell processes, and UEA lectin (Figure 22G and Figure 23H) produces negative labeling in the OB of the fox, as was the case in dog and wolf.

## 4. Discussion

The study of the anatomical diversity of the mammalian olfactory system has been defined by the existence of two major subsystems, the olfactory system and the vomeronasal system, both homologous in numerous morphological and functional features due to the extent that both systems present a degree of overlap in the stimuli they detect and the effects that they mediate [13,44]. However, the systems evolved in mammals following different pathways [45]. The olfactory system presents a general pattern of evolutionary conservation whose gene sequences are well-conserved, which is observed in its conserved morphological and neurophysiological traits and in the nature of the olfactory receptors, which specialized to detect a broad range of odors that remain stable in the environment. [46]. The vomeronasal system evolved to detect a limited group of species-specific ligands, primarily pheromones, which are the result of ecological adaptations closely related with the ecological context of each species. Morphologically, the VNS presents a high morphological diversity [47], and its VNO receptors are associated with a wide range of genes [48].

Perhaps for this reason, while there have been a large number of comparative morphofunctional studies of the vomeronasal system, there has been fewer in-depth studies of the neuroanatomical characteristics of the olfactory mucosa and the main olfactory bulb that take into account the specific and differential features of each species. This fact has meant that the study of the olfactory system has focused on a small number of models, which, although thoroughly explored, involves risk when making interspecies extrapolations [49].

An example of this is the study of the olfactory system of canids, collectively considered as macrosmatic animals par excellence [50], assuming an identical common organization without delving into the differential features between species within the family Canidae. Not only are comparative studies necessary to avoid the risks of extrapolation, but within this group we have the specific case of the domestication of the dog, an excellent model for the study of the effect of selection pressure on the structuring of the nervous system and the functional and molecular bases that have led in a short period of time to such important differences as those that differentiate the dog from its wild ancestors, the wolf and the fox.

One of the aspects that we have taken into account in our study is the macroscopic evaluation of the olfactory encephalon in the three species studied, observing in all the specimens an appreciably greater development of the olfactory bulbs in the wolf (Figure 9) and the fox (Figure 17) than in the dog (Figure 1). This is an observation that needs to be confirmed by morphometric and stereological studies, which faces the difficulty, especially in the case of the wolf, of obtaining samples in good condition from wild animals of similar age and of both sexes that can be compared with a representative sample of domestic dogs of a similar breed, age and conformation. However, our qualitative assessment is consistent with the known greater development of the wolf brain compared to the domestic dog brain [51,52].

In the case of the fox, the seminal study of selective breeding for tameness or aggression known as the Russian farm-fox experiment developed to understand the neural correlates of domestication has found that intense selection based on behavior can produce extremely rapid gross changes in distributed brain morphology [53]. In parallel to this, the comparison of endocranial volume between wild and domesticated foxes belonging to the Russian farm-fox experiment determined a reduction in endocranial volume development is a consequence of domestication [54]. These macroscopic observations on the effects of domestication on the brain have been correlated at the functional level with studies such as that by Bird et al. [32], suggesting that there is a loss of olfactory capacity in dogs compared to wild canids. Moreover, this study did not find evidence of any anatomical advantage in the nose of scent dogs, even in comparison to short-snouted dogs, supporting the hypothesis that scent breeds are scent breeds in name only. On the basis of these observations, we consider that the use in our study of canine individuals of mesaticephalic breeds, similar in conformation to the wolf, constitutes a valid approach. Finally, it is also significant to make reference to the specific study of the human species, where a direct correlation between the shape of the olfactory bulb and olfactory capacity has recently been described [55].

From a histological point of view, the three species show a wide and well-defined lamination, typical of a macrosmatic species, but there are important differences, the most outstanding being the development of the glomerular layer in the wolf and the greater cellularity of the olfactory bulb in the two wild canids studied. The development of the glomerular layer is particularly striking in the wolf (Figure 4, Figure 12 and Figure 19 for comparison), its glomerular distribution in terms of the number of rows of glomeruli (2–4) being similar to that found in elephants [56]. Of particular significance in this regard is the only morphological and neurochemical study in a wild canid of which we are aware, the one conducted on the African wild dog [29], which found that the glomerular diameter in this species was higher than that observed in the domestic dog and within the range observed in the African elephant [56]. With regard to the cellularity of the OB, the great development of the periglomerular cells and the large size of the mitral cells are striking in both wolves and fox (Figure 5, Figure 13 and Figure 20). The dendritic processes of the mitral cells observed in Nissl staining, especially in sagittal sections of the mitral layer, display much greater development in wolves and foxes than in dogs. The absence of morphological and stereological morphometric studies of the OB of canids does not allow these results to be contrasted, and opens the door to future stereological studies that should explore this aspect in greater depth.

As for immunohistochemical characterization of the OB, there are also no studies in wild canids to which we can compare our observations. To our knowledge, we can only cite the immunohistochemical study of calcium-binding proteins in dogs [28]. In this sense, our results are comparable to the labeling patterns obtained with anti-calbindin and anti-calretinin in the dog by the same authors. In wild canids, a more intense labeling pattern is observed for periglomerular and granular cells in the fox compared to the wolf, and likewise in the wolf compared to the dog. Although this is a descriptive rather than quantitative assessment, it is interesting to note that the aforementioned study by Choi et al. [28] directly correlates by Western blot the intensity of the immunolabeling observed with the levels of the calcium-binding proteins studied. As for the observations of Chengetanai et al. [29] in the African wild dog, this study only coincides with our work in terms of the immunomarkers used in the case of calcium-binding proteins, obtaining results similar to those observed in the wolf. With respect to other markers such as OMP, GFAP, Gao, Gai2 and MAP2, to our knowledge there are no antecedents in dogs and wild canids that allow us to contrast our results. Among these markers, the specific case of MAP-2 and GFAP, which produce a more intense response in both wild canids, are striking. MAP-2 expression is particularly relevant because it can be used to identify mitral cell dendritic trees [57,58]. Therefore, the strong labeling obtained with this marker in both the plexiform and glomerular layers of the wolf (Figure 15D) and fox (Figure 23D) is consistent with the histological observations of these cells. With respect to the somas of mitral cells, none of the markers allowed their characterization, which was also observed by Chengetanai et al. [29] with the markers they used in their study.

The histochemical study with the lectins LEA and UEA produced similar results in all three species. While LEA produces uniform labeling in the two superficial layers of the bulb (Figure 8I, Figure 15I and Figure 22H), UEA produces negative labeling (Figure 8H, Figure 15H and Figure 22G). Studies with LEA in a large number of species confirm this lectin as a universal marker for the mammalian olfactory system [59,60,61]. The case of UEA is more striking because this lectin, specific to L-fucose, is known to present a specific pattern towards the vomeronasal system in dogs [23] and foxes [34], while there was no information about it in wolves. Our results show that the specificity of the vomeronasal system towards the UEA is a highly conserved characteristic in the main olfactory system of canids. This is not the case in Felidae, as cat vomeronasal systems are not specific for UEA, similar to what happens in other groups of mammals as diverse as rodents [62], rabbits [63], pigs [64], ruminants [65] and marsupials [66].

## 5. Conclusions

Our study shows the existence of remarkable anatomical and histological differences between the olfactory bulb of dogs and that of the two wild canids studied: their most direct wild ancestors, the wolf and the fox. Further, at the morphofunctional level, the immunohistochemical pattern observed points to a higher degree of neurochemical activity in wild canids. A future step will be to carry out morphometric and stereological studies to quantitatively assess the morphological differences found.

## Figures and Tables

**Table 1:** Antibodies and lectins used, species of elaboration, dilution, catalogue number, manufacturer, target immunogens and relevant reference for each antibody, and RRID codes.

Antibody	1st Ab Species/Dilution	1st Ab Catalogue Number	Inmunogen	Reference	RRID	2nd Ab Species/Dilution, Catalogue No.
Anti-Gαo	Rabbit/1:200	Santa Cruz Biotech. sc-387	Peptide mapping within a highly divergent domain of rat Gαo	[37]	AB_2111641	ImmPRESS VR HRP Anti-rabbit IgG Reagent MP-6401-15
Anti-Gαi2	Rabbit/1:200	Santa Cruz Biotech. sc-7276	Peptide mapping within a highly divergent domain of rat Gαi2	[38]	AB_2111472	ImmPRESS VR HRP Anti-rabbit IgG Reagent MP-6401-15
Anti-OMP	Goat/1:400	Wako 544-10001	Rodent olfactory marker protein	[39]	AB_2315007	Horse anti-goat IgG 1:250 Vector BA-9500
Anti-CB	Rabbit/1:6000	Swant CB38	Rat recombinant calbindin D-28k	[40]	AB_10000340	ImmPRESS VR HRP Anti-rabbit IgG Reagent MP-6401-15
Anti-CR	Rabbit/1:6000	Swant 7697	Recombinant human calretinin with a 6-his tag at the N-terminus	[41]	AB_2619710	ImmPRESS VR HRP Anti-rabbit IgG Reagent MP-6401-15
Anti-MAP-2	Mouse/1:400	Sigma M4403	Rat brain microtubule-associated proteins	[42]	AB_477193	ImmPRESS VR HRP Anti-mouse IgG Reagent MP-6402-15
Anti-GFAP	Rabbit/1:400	Dako Z0334	GFAP from bovine spinal cord	[43]	AB_10013382	ImmPRESS VR HRP Anti-rabbit IgG Reagent MP-6401-15
UEA	1:60	Vector L-1060				Rabbit 1:50 DAKO P289
LEA	20 µm/mL	Vector B-1175				Vectastain ABC reagent PK-4000

## Data Availability

All relevant data are within the manuscript, and are fully available without restriction.
